# Total pancreatectomy and pancreatic fistula: friend or foe?

**DOI:** 10.1007/s13304-021-01130-3

**Published:** 2021-08-07

**Authors:** Roberto Salvia, Gabriella Lionetto, Giampaolo Perri, Giuseppe Malleo, Giovanni Marchegiani

**Affiliations:** Department of Surgery, Dentistry, Gynecology and Pediatrics (DSCOMI), Unit of General and Pancreatic Surgery, University of Verona, G.B. Rossi Hospital, P.Le Scuro 10, 37134 Verona, Italy

**Keywords:** Total pancreatectomy, Pancreatic fistula, Completion pancreatectomy, High-risk pancreas

## Abstract

Postoperative pancreatic fistula (POPF) still represents the major driver of surgical morbidity after pancreaticoduodenectomy. The purpose of this narrative review was to critically analyze current evidence supporting the use of total pancreatectomy (TP) to prevent the development of POPF in patients with high-risk pancreas, and to explore the role of completion total pancreatectomy (CP) in the management of severe POPF. Considering the encouraging perioperative outcomes, TP may represent a promising tool to avoid the morbidity related to an extremely high-risk pancreatic anastomosis in selected patients. Surgical management of severe POPF is only required in few critical scenarios. In this context, even if anecdotal, CP might play a role as last resort in expert hands.

**Supplementary Information:**

The online version contains supplementary material available at 10.1007/s13304-021-01130-3.

## Introduction

Pancreatic surgery represents a complex surgery, still burdened by high morbidity rates despite advances in surgical technique and perioperative care. Postoperative pancreatic fistula (POPF) remains the major driver of surgical morbidity after pancreaticoduodenectomy (PD). Grade C POPF per the International Study Group of Pancreatic Fistula (ISGPF) definition [1]—i.e., life-threatening fistulae mostly requiring a surgical re-intervention—has an estimated incidence of 2% with a mortality reaching 35% [2]. Predictive factors and appropriate management strategies for this dreadful complication are still under scrutiny.

Efforts to prevent severe POPF have often focused on the surgical technique of the pancreatic anastomosis, either a pancreatico-jejunostomy (PJ) or a pancreatico-gastrostomy (PG) with or without stenting [3]. This is particularly true in the high-risk pancreas scenario, where the long-term benefits of pancreatic stump preservation (i.e., lesser incidence of exo-endocrine insufficiency) are mirrored by harsher postoperative courses [4]. Other experience had addressed the same dilemma. In a prospective study, Mazzaferro et al. investigated the safety and efficacy of pancreatic duct occlusion with neoprene-based glue in patients undergoing PD at high risk of POPF. They reported that pancreatic duct occlusion equalizes short-term postoperative outcomes to those patients at lower risk of POPF with, however, a three-fold higher risk of post-surgical diabetes [5].

In this perspective, the encouraging postoperative outcomes of total pancreatectomy (TP) reported at high-volume centers in recent years [6, 7] have led the authors questioning whether the burden of complications related to a high-risk pancreatic anastomosis could justify the use of TP as an alternative strategy in selected patients to avoid the occurrence of POPF [8].

Conversely, when a POPF has already developed after PD, salvage surgery may be required after failure of non-operative strategies [1]. Different intra-operative procedures have been reported, including completion pancreatectomy (CP). CP is a technically demanding operation performed often in critically ill patients due to life-threatening sepsis and/or bleeding, and therefore characterized by a strikingly high mortality (ranging up to 80%) [9].

The aim of this review is to critically analyze current evidence supporting the use of TP to prevent POPF in the setting of a high-risk pancreas (a promising new strategy?) and, on the other end, to review the role of CP in the management of severe POPF (the swansong of an obsolete operation?).

## Total pancreatectomy to avoid pancreatic fistula in high-risk patients: a promising new friend?

Several risk score systems based on pre- and intra-operative parameters, such as the Fistula Risk Score (FRS) [10] or the alternative Fistula Risk Score (a-FRS) [11], have been proposed to predict the occurrence of POPF and stratify patients based on this risk.

The most validated of these scores is the FRS (0–10 points), calculated at the time of pancreatic anastomosis after the pancreatic head resection, on the basis of the weighted influence of 4 risk factors: (1) pancreatic stump texture (firm vs soft); (2) disease pathology (low vs high risk); (3) pancreatic duct size and (4) estimated intra-operative blood loss (Table 1). This score identifies a distinct high-risk cohort (FRS 7–10), which represents around 10% of all PDs and shows substantially worse clinical outcomes, including a CR-POPF rate approaching 30% (and up to 100% in case of FRS 9–10) [4].

**Table 1:** Fistula risk scoring system according to Callery et al. [10]

Risk factor	Parameter	Points
Pancreatic stump texture	Firm	0
	Soft	2
Pathology	Low risk (PDAC, chronic pancreatitis)	0
	High risk (ampullary, duodenal, cystic, pNET, etc.)	1
Pancreatic duct size (mm)	≥ 5	0
	4	1
	3	2
	2	3
	≤ 1	4
EBL (ml)	≤ 400	0
	401–700	1
	701–1000	2
	> 1000	3
		Total: 0–10

*EBL* estimated blood loss, *PDAC* pancreatic ductal adenocarcinoma, *pNET* pancreatic neuroendocrine tumor

Despite the ability to stratify the POPF risk, opinions and controversies upon prevention, mitigation and treatment strategies in high-risk pancreas continue to fuel the debate among surgeons worldwide.

In a recently published randomized trial [12], our group failed to assess superiority of PJ reconstruction over PG for the prevention of POPF in high-risk pancreas. However, the decreased rate of Clavien–Dindo ≥ 3 (23 vs 47%) and the lower average complication burden associated with PJ with externalized stent in our experience seems to justify its adoption in this setting. In the era of mitigation strategies [4], this latter finding seems particularly convincing, even when considering the worrisome outcomes related to the externalized stent malfunction, an event occurring in about one-fifth of reported cases and associated with a significantly increased rate of POPF and its severity grade [13].

As mentioned above, recent studies have reported improved perioperative outcomes and postoperative quality of life after TP, presumably due to centralization at high-volume centers and development of long-acting insulin and modern pancreatic enzyme preparations [6, 7, 14, 15].

This has led our group to retrospectively compare short- and long-term outcomes of TP vs PD in patients at high risk for POPF development. Indeed, patients undergoing TP exhibited lower rates of major morbidity (19 vs 31%) and a comparable mortality (3 vs 4%). Despite these promising postoperative outcomes, performing TP still raises important concerns due to the inevitable presence of its long-term sequelae. In fact, although general, cancer- and pancreas-specific quality of life were comparable between the high-risk PD and TP groups, pancreatic insufficiency affected more severely TP patients with a 100% endocrine and exocrine insufficiency rate, compared to only 13% and 63% in the high-risk PD patients, respectively. Moreover, TP patients showed worse diabetes-related quality-of-life impairment.

Similarly, Capretti et al. reported favorable short-term outcomes in a retrospective cohort of high-FRS patients undergoing intra-operative CP as an alternative to performing a high-risk pancreatic anastomosis. Notably, decision to perform a TP was often made in older patients with higher ASA score, higher BMI and pre-existing diabetes. The finding of an overall lower complication rate (with a similar—though not significant—trend in major morbidity) in the CP group, corroborated by the absence of major long-term adverse events related to the pancreatic insufficiency, led the authors to consider TP in a selected group of patients, namely those at higher risk of failure to rescue from POPF and those in whom the impact would might be less relevant in the long term [16].

**Fig. 1:**
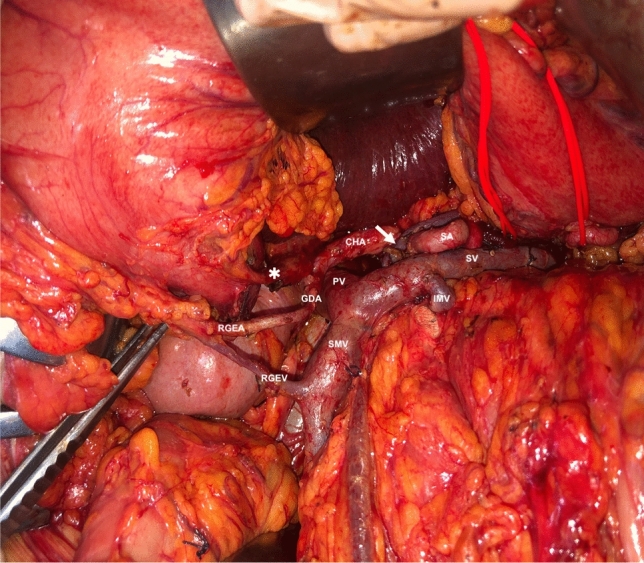
Operative field after TP with spleen and vessels preservation. The white arrow indicates the left gastric vein, the asterisk the right gastric artery. In this case also, the gastroduodenal artery (GDA), the right gastroepiploic artery (RGEA) and vein (RGEV) were preserved. *CHA* common hepatic artery, *IMV* inferior mesenteric vein, *PV* portal vein, *SMV* superior mesenteric vein, *SA* splenic artery, *SV* splenic vein

**Fig. 2:**
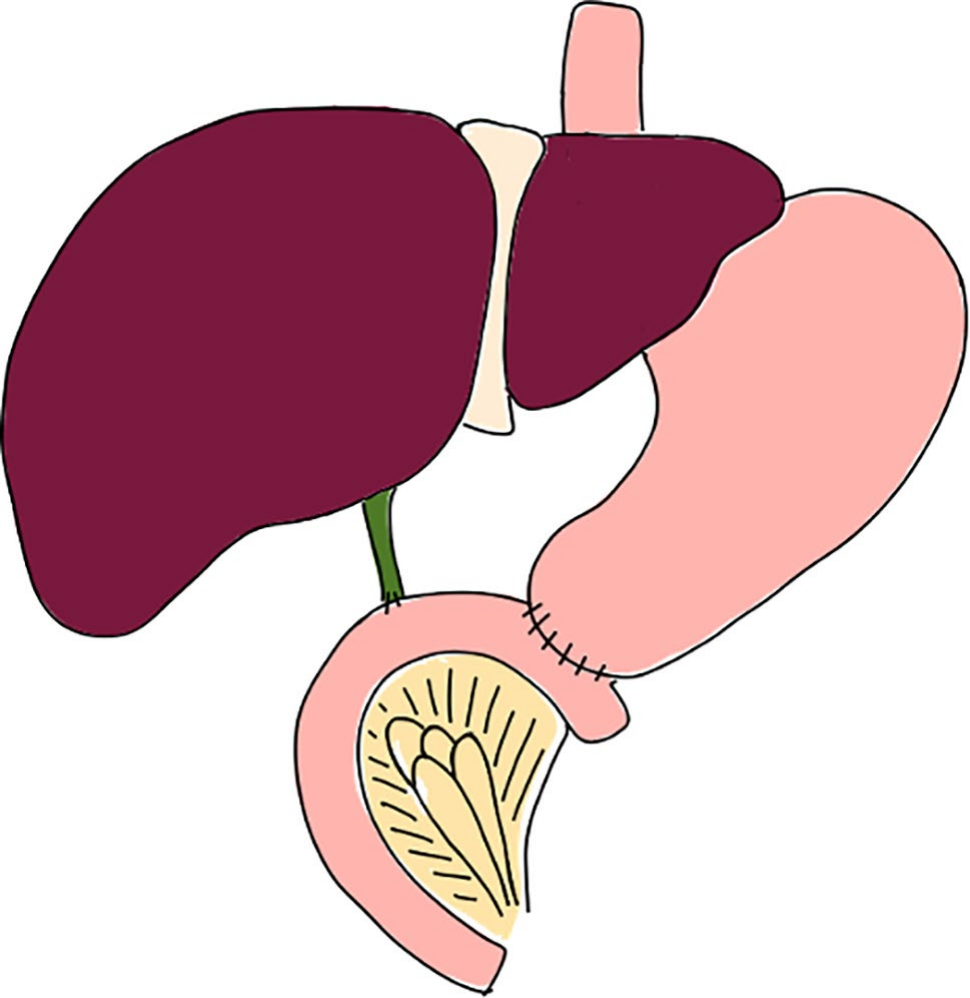
Reconstruction after TP with a single jejunal loop with duodenojejunostomy followed by hepaticojejunostomy

Another possible concern following TP is represented by gastric complications, rarely reported in surgical series, but potentially responsible of severe morbidity and mortality [17]. Indeed, TP necessitates ligation of the right gastric and gastroepiploic veins. Association with splenectomy would impair also the left venous drainage (i.e., splenic vein, left gastroepiploic vein and short gastric vessels), leaving just the coronary vein and the esophageal plexus as major route of gastric venous outflow. Therefore, left gastric vein and spleen preservation must be considered—if oncologically feasible—to preserve the gastric venous outflow and avoid the risk of gastric venous congestion, which may lead to mucosal erosions and hemorrhage and, ultimately, to gastric necrosis and perforation. Furthermore, it is possible to speculate that attempts to preserve the arterial gastric inflow—namely the right gastric artery—could reduce the risk of ischemic complications, considering that usually after a “standard” TP only the left gastric artery provides the global arterial inflow. In this regard, a pylorus-preserving TP with reconstruction by a single jejunal loop with duodenojejunostomy with preservation of right gastric artery followed by hepaticojejunostomy may be effective to minimize the risk of devascularization linked to stomach mobilization (Fig. 1), and also to allow a feasible endoscopic access to manage both the anastomosis in case of related complications and sequelae (Fig. 2).

Unfortunately, data exploring this topic are sparse and come solely from recently published studies [8, 16]. Far from advocating the systematic use of TP in high-risk scenarios, we think that it may be considered as an alternative to perform a high-risk pancreatic anastomosis in highly selected patients for whom the short-term benefits in the postoperative setting may overcome the disadvantages due to the complete absence of residual pancreatic function. For example, it might play a role in oncological patients, for whom access to adjuvant chemotherapy, which is often delayed by the occurrence of POPF [2], could be crucial.

Thus, we are now facing the delicate ethical implications of this decision as we aim to test our hypothesis in a randomized controlled setting. Patients who are deemed to undergo a high-risk anastomosis, after a “pancreas-specific” risk assessment considering main pancreatic duct and pancreatic remnant characteristics, will be sorted to receive either a “standard” reconstruction or a completion TP. Postoperative outcomes will be compared together with pancreatic insufficiency and long-term quality of life.

## Total pancreatectomy to treat postoperative pancreatic fistula: the swansong of an obsolete foe?

Over the last decades there has been a shift from operative to non-operative management of POPF [18]. Although the majority of POPF can be managed with conservative therapy, some do require surgical intervention either because of inaccessibility of infected collection to percutaneous or endoscopic drainage, or because of clinical instability associated with uncontrolled sepsis and multi-organ failure.

To our knowledge, only a systematic review addressed the role of CP in the acute management of fistula [9]. However, despite the heterogeneity between surgical series—all retrospective with inclusion period spanning over two decades—the huge mortality rate stands out.

A systematic search was performed, to provide a broad perspective of the strikingly high mortality of this nowadays rarely performed procedure. A flowchart showing the selection process is available in the Annex. Mortality rates of all major surgical series exploring CP for POPF are outlined in Table 2. Whether this dismal scenario is linked to the surgical burden itself versus the delay of intervention is debatable.

**Table 2:** Overview of major series reporting mortality after CP for POPF management

Authors (year)	Period	Number of PD	Incidence of POPF n (%)	Relaparotomy due to POPF n (%)	CP n (%)	Mortality after CP (%)
Garnier (2021) [19]	2012–2019	450	77 (17.1)	30 (6.7)	21 (4.7)	23.8
Luu (2020) [20]	2007–2016	722	125 (17.3)	23 (3.2)	19 (2.6)	36.8
Wronski (2019) [21]	2003–2017	616	67 (10.9)	43 (7.0)	17 (2.8)	47.1
Nentwich (2015) [22]	2002–2012	521	NA	NA	20 (3.8)	55.0
Almond (2014) [23]	1987–2013	1232	NA	NA	38 (3.1)	52.6
Balzano (2014) [24]	2004–2011	669	201 (30.0)	37 (5.5)	14 (2.1)	21.4
Ribero (2013) [25]	1990–2010	370	112 (30.3)	47 (10.8)	23 (6.2)	43.4
Paye (2013) [26]	2005–2011	254	NA	21 (8.2)	4 (1.6)	50.0
Govil (2012) [27]	1999–2006	208	NA	12 (5.8)	2 (0.9)	50.0
Xu (2010) [28]	1984–2009	963	103 (10.7)	12 (1.2)	5 (0.5)	20.0
Fuks (2009) [29]	2000–2006	680	111 (16.3)	36 (5.3)	2 (0.3)	50.0
Haddad (2009) [30]	2000–2006	117	35 (29.9)	14 (12.0)	5 (4.3)	40.0
Bachellier (2008) [31]	1988–2005	403	NA	12 (2.9)	8 (2.0)	50.0
Müller (2006) [32]	2001–2006	NA	NA	NA	23 (NA)	39.1
Tamijmarane (2006) [33]	1987–2005	599	NA	NA	23 (3.8)	56.5
de Castro (2005) [34]	1992–1996	459	41 (8.9)	NA	9 (2.0)	0.0
Gueroult (2004) [35]	1989–1999	282	38 (13.5)	NA	8 (2.8)	37.5
Schlitt (2002) [36]	1988–2000	441	33 (7.5)	29 (6.6)	10 (2.3)	80.0
van Berge (1998) [37]	1983–1995	269	29 (10.8)	NA	8 (3.0)	0.0
Farley (1996) [38]	1972–1994	458	NA	NA	17 (3.7)	23.5
Cullen (1994) [39]	1980–1992	375	66 (17.6)	18 (4.8)	7 (1.9)	71.4
Smith (1992) [40]	1964–1988	479	NA	NA	11 (2.3)	63.6

*PD* pancreaticoduodenectomy, *POPF* postoperative pancreatic fistula, *CP* completion pancreatectomy, *NA* non-available

Indications to CP are not uniform and depend mostly on a critical patient’s fitness for an operation with a median duration ranging from 144 to 240 min and reported blood loss of 900–2500 ml [9] Generally, as the median time between elective surgery and CP ranges between 6 and 17 days [9], surgeons have to deal with a surgical field hindered by severe pancreatitis and inflamed surrounding adjacent abdominal organs, where even to get the access to the complication site is a highly demanding procedure. For this reason, CP is to be considered only in the hands of extremely experienced pancreatic surgeons.

Garnier et al. advocate for earlier threshold for re-exploration in high-risk patients, speculating that in this setting the advantage of performing a CP d’emblée when a pancreatic dehiscence is documented outweighed the resulting exocrine and endocrine insufficiency, given that at least the patient would be alive. Of note they reported lower blood loss rate probably due to the possibility of preserving the spleen in 43% of the cohort. Unfortunately, given the heterogeneity of scenarios in which CP may be required, it is difficult to reach a level of evidence higher than retrospective case-series in this regard (see Table 2).

While we agree that there might be a very selected pool of patients who could benefit from early CP, how to identify them remains nebulous. In our current practice, this operation is exceedingly rarely performed, and reserved for cases in which all other options have been exhausted. Considering the worldwide trends towards a minimally invasive and conservative approach, nowadays it is not difficult to imagine how CP may be more and more relegated to a last resort in critical scenario.

## Conclusion

Considering the encouraging perioperative outcomes, TP may represent a promising “ally” to avoid the morbidity related to a high-risk pancreatic anastomosis in highly selected patients, although important differences in the long-term quality of life remains a major concern to be explored.

Surgical management of POPF is mainly based on surgeon know-how and gut-feeling in an almost always “desperate” situation. In this context, even if anecdotal, CP might sometimes play a role. The best timing for CP, however, still remains a matter of speculation.

## Supplementary Information

Below is the link to the electronic supplementary material.

Supplementary file1 (DOCX 24 KB)
