# Supplementary material for: Total pancreatectomy and pancreatic fistula: friend or foe?
Source: Updates Surg. 2021 Aug 7;73(4):1231–6. doi: 10.1007/s13304-021-01130-3 (PMC8397676; doi:10.1007/s13304-021-01130-3)
Supplement: Supplementary file 1 — Supplementary file1 (DOCX 24 KB) [file 13304_2021_1130_MOESM1_ESM.docx]

**Supplementary Table 1** Flowchart of articles through selection process. A systematic search was completed of the PubMed and EMBASE databases for all studies reporting mortality after CP for pancreatic fistula published from January 1964 until February 2021. Combinations of the following keywords were used: ("pancreaticoduodenectomy" or "completion pancreatectomy" or "total pancreatectomy") AND ("fistula" or "leak"). References lists of selected articles were also evaluated for additional publications.

Records identified through database searching

(n=1987)

Records screened

(n=1993)

Records identified through reference lists scrutiny

(n=6)

)

Full-text articles assessed for eligibility

(n=26)

Full-text articles excluded (n=4)

No clinical outcomes (n=1)

Population overlapping (n=1)

Review articles (n=2)

Studies included

(n=22)

Records excluded

(n=1967)
